# Dacryoendoscopy-guided re-canalization of canaliculops: Two case reports

**DOI:** 10.1097/MD.0000000000024985

**Published:** 2021-03-12

**Authors:** Tomoko Kitada, Masashi Mimura, Yasuhiro Takahashi, Mai Takagi, Hidehiro Oku, Tsunehiko Ikeda

**Affiliations:** a Department of Ophthalmology, Osaka Medical College, Takatsuki; b Department of Ophthalmology, Osaka Kaisei Hospital, Osaka; c Department of Oculoplastic, Orbital & Lacrimal Surgery, Aichi Medical University Hospital, Nagakute, Aichi, Japan.

**Keywords:** bicanalicular intubation, canaliculops, case report, dacryoendoscope

## Abstract

**Rationale:**

Canaliculops is a rare condition, and only 11 cases have been reported previously. We report 2 cases of canaliculops, which were successfully treated using the new recanalization technique under dacryoendoscopy followed by bicanalicular lacrimal intubation.

**Patient concerns:**

A 78-year-old man and a 76-year-old woman had 3- and 1-year histories of medial-upper eyelid swelling (left and right, respectively) without any inflammatory signs, history of periocular trauma, herpes infection, use of specific drugs, or ophthalmic diseases of note.

**Diagnoses:**

The cystic lesions were evaluated using ultrasound biometry or computed tomography to find the lumen of the horizontal canaliculus was exceedingly expanded, and to confirm the clinical diagnosis of canaliculops.

**Interventions:**

As the 2 cases of canaliculops were caused by upper puncta and common canaliculus obstructions, canaliculops of the upper eyelid were recanalized under dacryoendoscopic guidance, followed by bicanalicular intubation. The tubes were kept in situ involving bi-weekly irrigation and instillation of antibiotic and anti-inflammatory eye drops, and were removed after 2 to 3 months of follow-up.

**Outcomes:**

Epiphora, and eyelid swelling were completely resolved immediately after the procedure, and the lesions did not recur on follow-up after more than 6 months.

**Lessons:**

Eleven case series of canaliculops have been described previously, but this is the first report of this recanalization procedure offering a new, less invasive treatment option compared to complete or partial resection of the cystic lesion.

## 1 Introduction

Canaliculops is a tense, bluish cystic lesion found in the medial canthus, characterized as a non-inflammatory ectasia of the lacrimal canaliculus filled with non-infectious serous fluid and mucus. This is a rare condition, and only 11 cases have been reported previously. Those cases showed a female predominance as well as primary lacrimal drainage obstruction, and the pathology can occur in upper or lower canaliculi, or even both as described in 1 case.^[1–7]^ Although the causes remain unclear, trauma, herpetic infections, or chronic drug use have been suggested as possible contributing factors.^[5]^ Canaliculops causes epiphora and cystic eyelid swelling, for which complete or partial resection of the cystic lesion, marsupialization of the ectasia, and re-canalization without the use of imaging studies are diagnostic treatment options.^[3]^ However, these procedures have low reliability and high invasiveness.

Herein, we report 2 cases of successful recanalization of canaliculops under dacryoendoscopy followed by lacrimal intubation. This technique seems more reliable and less invasive, suggesting a new alternative to previously reported procedures.

## 2 Report of cases

This study was conducted in accordance with the tenets of the Declaration of Helsinki and its later amendments. Written informed consent for publication was obtained from both patients.

This retrospective, observational case series included a 78-year-old man (involving the left eye) and a 76-year-old woman (involving the right eye). None of the patients had any history of periocular trauma, herpes infection, use of specific drugs, or ophthalmic diseases.

Both patients showed a cystic mass in the medial upper eyelid. The upper punctum was obstructed, resulting in a high-tear meniscus. The lower punctum, lower lacrimal canaliculus, lacrimal sac, and nasolacrimal duct were patent, as confirmed by lacrimal irrigation. The mass in both patients was clinically diagnosed as canaliculops and was treated by recanalization under dacryoendoscopic guidance, followed by bicanalicular intubation. All surgeries were performed under local anesthesia in an office setting by 2 of the authors (MM and YT).

We present the details of each case as below.

### 2.1 Presentation of case 1

**Figure 1:**
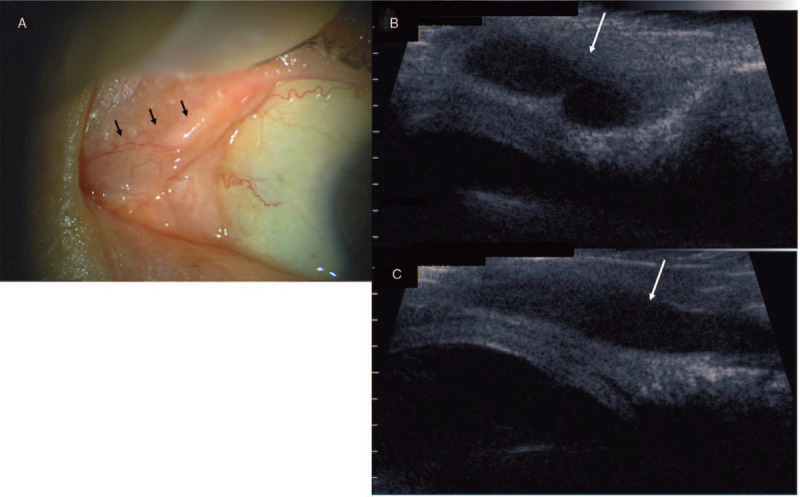
Serial images of the canaliculops in the left eye of a representative case. (A) Clinical photograph of the cystic mass at the medial upper eyelid (arrows) showing no evidence of inflammation. (B) Cross-sectional imaging using an ultrasound bio-microscope showing gourd-shaped ectasia of the canaliculus. (C) Horizontal section showing expansion of the entire canaliculus, about 9 mm in length, without any evidence of tumor, foreign body, or dacryolith.

A 78-year-old man became aware of the left epiphora and swelling of the left upper eyelid 3 years before referral. At the initial presentation, a cystic mass was found at the medial end of the left upper eyelid without hyperemia or pain (Fig. 1A). Imaging using ultrasound biomicroscopy (UBM) at 30 MHz showed a well-defined, gourd-shaped cyst without any evidence of foreign bodies or dacryolith migration (Fig. 1B and C). We thus diagnosed canaliculops and proceeded with re-canalization of the upper canaliculus using dacryoendoscopy.

**Figure 2:**
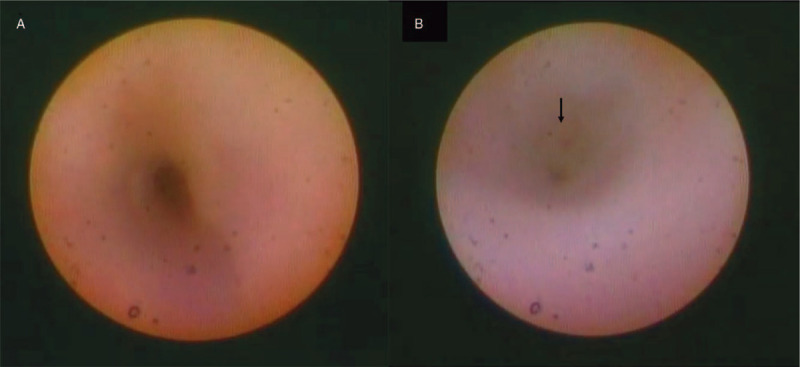
Serial images of dacryoendoscopy showing the inner lumen of the canaliculops in the representative case. (A) The wall is smooth and clean without any evidence of inflammation. (B) Obstruction of the common canaliculus can be seen at the end of the cystic lesion (arrow).

**Figure 3:**
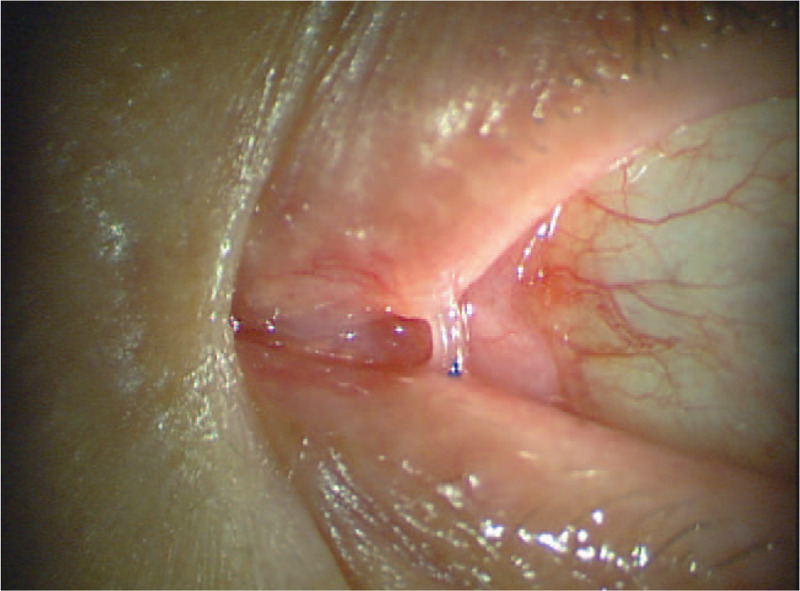
Clinical photographs of the canaliculops in the representative case, immediately after lacrimal intubation. Note the cyst appeared deflated compared to that preoperatively shown in Figure 1A.

After local infiltration of anesthesia around the cyst using 1% lidocaine with 1:100,000 epinephrine, punctoplasty of the upper punctum was performed to insert the dacryoendoscope into the canaliculus. Although the lumen of the vertical canaliculus was slightly dilated, that of the horizontal canaliculus was exceedingly expanded (Fig. 2A). The inner wall of the cyst was lined with smooth, whitish mucosa, and the lumen was filled with transparent serum contents, resulting from minor inflammatory changes in the cyst. The cyst ended in the common canaliculus (Fig. 2B). The upper canaliculus was recanalized using sheath-guided endoscopic probing (SEP), followed by bicanalicular intubation under dacryoendoscopic guidance. The dacryoendoscope was loaded into a “sheath” made by an 18-gauge infusion catheter, and the composite was inserted into the upper punctum to puncture the obstructed point in the common canaliculus. The composite was then pushed and advanced through the lacrimal sac and nasolacrimal duct into the nasal cavity. The dacryoendoscope was removed while leaving the sheath temporarily in place within the lacrimal duct. One side of a bicanalicular tube was inserted into the sheath, followed by removal of the sheath from the nasal cavity. The same procedure was performed from the lower punctum to complete bicanalicular intubation. Immediately after surgery, the cyst appeared deflated (Fig. 3).

Postoperative care involved bi-weekly irrigation and instillation of antibiotic and anti-inflammatory eye drops. The lacrimal tube was removed 2 months postoperatively. All symptoms resolved without recurrence for more than 1 year postoperatively.

### 2.2 Presentation of case 2

A 76-year-old woman became aware of right upper eyelid swelling 1 year before referral. At the initial presentation, slit-lamp examination demonstrated a bluish cystic mass without any evidence of inflammation at the medial end of the left upper eyelid. Computed tomographic images showed a mass, but this mass did not seem to extend to the lacrimal sac fossa. Based on these findings, a diagnosis of canaliculops was made. The same surgical procedure as in case 1, recanalization and bicanalicular intubation under dacryoendoscopy, was performed, followed by the tube kept in situ for 3 months, to the successful outcome without recurrence at the 1-year follow-up period.

## 3 Discussion

The current case series reports 2 cases of canaliculops that were successfully treated by recanalization and lacrimal intubation under dacryoendoscopy.

Canaliculops represent ectasia of the canaliculus resulting from obstruction of both the punctum and common canaliculus, similar to dacryocystocele. Previous case reports have recommended complete or partial excision and marsupialization of the cyst wall for the treatment of canaliculops.^[3]^ However, these procedures are invasive and can pose a risk of re-obstruction of the lacrimal canaliculus. Conversely, recanalization of the lacrimal canaliculus under dacryoendoscopy does not require excision of the cyst wall. In addition, dacryoendoscopy enables reliable probing of the obstructed point at the common canaliculus and insertion of a tube under direct visualization, even in cases where the ectasia obscures the end of the cyst, which impedes accurate probing. Subsequent bicanalicular intubation is advantageous in terms of its stability and high success rate, particularly for common canalicular obstruction (90.1%–94.6%),^[8,9]^ which could lead to successful long-term outcomes.

On the other hand, pathological specimens cannot be harvested in our procedure. A lining of nonkeratinized multilayer original squamous epithelium in the cyst wall, superficial cytokeratin 7 staining, and patchy suprabasal cytokeratin 17 staining are significant pathological findings for the diagnosis of canaliculops.^[1,6]^ However, dacryoendoscopy can directly detect mucosal abnormality, ectasia of the lacrimal canaliculus, and obstruction of the common canaliculus, allowing sufficient clinical diagnosis of canaliculops without histopathological examination.

Tumors, foreign bodies, and other space-occupying lesions, such as dermoid cysts, mucoceles, and meningoencephalocele, are included as differential diagnoses for canaliculops. Computed tomography, as used in 1 of our patients, or magnetic resonance imaging can provide additional information on the lesion, particularly for large lesions. However, low-resolution images make differential clinical diagnosis difficult. UBM provides higher-resolution images of canaliculops,^[5]^ particularly the interior of the distended area. This is more useful for the differential diagnosis of tumors and foreign bodies, including dacryoliths, preoperatively.

In conclusion, we have reported 2 rare cases of canaliculops treated successfully by recanalization using dacryoendoscopy. Based on previous reports on canaliculops with histopathological arguments, we suggest that less invasive recanalization utilizing lacrimal dacryoendoscopy can be a considerable intervention for canaliculop patients.

## Author contributions

**Conceptualization:** Masashi Mimura, Yasuhiro Takahashi.

**Data curation**: Tomoko Kitada, Masashi Mimura, Yasuhiro Takahashi, Mai Takagi.

**Investigation**: Tomoko Kitada, Masashi Mimura, Yasuhiro Takahashi, Mai Takagi.

**Methodology**: Masashi Mimura, Yasuhiro Takahashi, Mai Takagi.

**Supervision:** Hidehiro Oku, Tsunehiko Ikeda.

**Writing – original draft:** Tomoko Kitada, Masashi Mimura.

**Writing – review & editing:** Yasuhiro Takahashi, Hidehiro Oku, Tsunehiko Ikeda.
